# Transcriptome-Wide Analysis of Neutrophil-Related Circ_22232 in Neuroinflammation from Ischemic Stroke Mice

**DOI:** 10.3390/brainsci13091283

**Published:** 2023-09-04

**Authors:** Zheng Sun, Youdong Zhou, Yanting Liu, Ran Luo, Chunlei Tian, Qianxue Chen

**Affiliations:** 1 Department of Neurosurgery, Renmin Hospital of Wuhan University, Wuhan 430060, China; szyczxyy@163.com; 2 Department of Neurosurgery, Yichang Center People’s Hospital, Yichang 443003, China; hanningzi@126.com (Y.Z.); yczxyylyt@163.com (Y.L.); lrns2005@163.com (R.L.)

**Keywords:** ischemic stroke, circ_22232, neuroinflammation, neutrophil

## Abstract

Ischemic stroke (IS) often leads to high rates of disability and mortality worldwide with secondary damage due to neuroinflammation. Identification of potential therapeutic targets via the novel circular RNAs (circRNAs) would advance the field and provide a better treatment option for neuroinflammation after IS. Gene Ontology Term Enrichment (GO) and Kyoto Encyclopedia of Genes and Genomes (KEGG) were applied to identify differentially expressed genes/miRNAs/circRNAs in the genome-wide RNA-seq profiles of ischemic mice. Meanwhile, relevant circRNAs were screened by differential expression analysis and coexpression RNA regulation network analysis. To explore the function of circ_22232 (Specc1l), we generated circ_22232 knockdown mice and applied middle cerebral artery occlusion (MCAO) to study IS. Cytokine levels were detected by enzyme-linked immunosorbent assay. Morphological changes were observed with immunohistochemical staining and hematoxylin-eosin staining. The circ_22232/miR-847-3p/Bmp1 axis was found to be highly correlated with neutrophil-associated neuroinflammation in cerebral tissue of mice. Immunohistochemical showed a progressive increase in the proportion of neutrophils after IS. In in vivo experiments, the circ_22232 knockdown alleviated cerebral injury by reducing the activation of neutrophils and inflammatory cytokine production. This suggests that circ_22232 is associated with inflammation, which may serve as a potential therapeutic target for IS.

## 1. Introduction

Ischemic strokes (IS) frequently lead to serious disability and death on a global scale [1], which is typically associated with an inflammation response [2]. It is widely accepted that the prognosis of IS is strongly related to the status of the inflammation response. However, the existing treatments, such as thrombectomy and thrombolysis, do not always improve the prognosis well. Therefore, there has recently been much more attention paid to post-stroke inflammation as a novel treatment [3,4]. In the early stages of IS, inflammatory processes are temporarily elevated and provide some protection, but when inflammatory stimuli increase, a series of inflammatory cascades are triggered, causing the secondary damage and dysfunction [5,6]. Therefore, immunomodulation-based inflammation management may reduce brain damage and improve prognosis. Unfortunately, there are no effective treatments for primary injury after IS.

Recent gene sequencing and comprehensive studies of the genome-wide transcriptome have shown that noncoding RNAs (ncRNAs), including circRNAs, are altered in the progression of ischemic stroke by mediating apoptosis, autophagy, oxidative stress, inflammatory responses, angiogenesis, and disruption of the blood–brain barrier (BBB). A massive stroke mouse brain exhibits significantly changed in circRNA expression profiles [7,8]. Other clinical studies have demonstrated significant differences in the expression profiles of circ RNA between IS patients and healthy controls [9,10], suggesting that circRNA may be involved in the formation of IS; however, its mode of action is not yet understood.

The inflammatory response, which is prominent at all stages of cerebral ischemia injury, is one of the key pathogenic mechanisms of IS. During cerebral ischemia, microglia, chemotactic leukocytes, and inflammatory cytokines may be activated and secreted, aggravating damage to brain neurons. CircRNAs may regulate signaling or gene expression to support the inflammatory response associated with IS. However, related studies into circRNAs have only ever been performed in IS, which mainly targeted to the role of microglia in inflammation response. To provide novel targets for IS, our study will use bioinformatics to look at neutrophil-related circRNAs in neuroinflammation.

## 2. Materials and Methods

### 2.1. Genome-Wide Transcriptomic Data Processing

Annotation files associated with the expression profiles of GSE46266 (miRNA), GSE131193 (mRNA), and GSE196448 (cirRNA) were downloaded from the GEO database (https://www.ncbi.nlm.nih.gov/geo/, accessed on 8 January 2023). The miRNA data contained a total of 9 groups, including 4 normal controls, which were obtained from the brain tissue of mice, 2 at day 1 post-MCAO group, and 3 groups at 3, 5, and 7 days post-MCAO. The mRNA data contained a total of 12 groups, which were obtained from the brain tissue of mice, including 3 normal controls and 1 and 7 days post-MCAO groups. The circRNA data included a total of 9 groups, which were obtained from the brain tissue of mice, including 3 normal controls and 3 groups at 3 and 7 days post-MCAO. The row stack of the expression matrix was modified with the limma package and annotated with the tidyverse package. The block diagram we designed is shown in Figure S1.

### 2.2. Screening of Differentially Expressed Genes (DEGs)

DEGs analyses and normalization were conducted by the Deseq2 R package [11]. Duplicate genes were removed, and the mean values were used as expression values. Absolute log2 fold change ≥ 1.5 were considered significant [12]. All q-values were corrected for statistical significance in multiple groups using the false discovery rate (FDR) (*p* < 0.05, FDR < 0.05).

### 2.3. Functional and Pathway Enrichment Analyses

The Bioconductor R package cluster Profiler was used to explore the functional annotations of Genomes, Gene Ontology Term Enrichment (GO), and Kyoto Gene Encyclopedia (KEGG) [12]. GO was used to explore the functions of genes in terms of molecular function (MF), biological process (BP), and cellular component (CC). KEGG was applied to perform pathway analysis, and the cutoff value of *p* < 0.05 was considered significant. ClueGO plugin [13] and Cytoscape software [14] were used to build an interaction network for BP terms. The STRING function of the protein association network was used to analyze the protein–protein interaction (PPI) (https://string-db.org, accessed on 10 January 2023).

### 2.4. CircRNA-miRNA-mRNA Coexpression Network

The Starbase database (https://starbase.sysu.edu.cn/starbase2/index.php, accessed on 12 January 2023) was applied to predict the correlation of circRNA-miRNA and mRNA-miRNA. The cystoscope software was used to plot the gene regulatory network of circRNA, mRNA, and miRNA. We obtained a list of mRNA genes controlling neutrophil activation from the MSigDB website (https://www.gsea-msigdb.org/, accessed on 12 January 2023) and calculated the correlation between these genes and the above network-related functional genes. Seq-immuCC [15] was used to assess the relative percentage of different immune cell subtypes in the samples.

### 2.5. Animals

The male four-week-old C57BL/6J mice were gained from Shulaibao Biotechnology Co. Ltd. (Wuhan, China). Mice were housed in a constant humidity and temperature environment with free access to water and food. All animal experiments were conducted on a specific pathogen-free experimental platform at the Experimental Animal Center of the Renmin Hospital, Wuhan University (Wuhan, China). To perform the randomization, rats were randomly allocated into different groups. No significant differences between the weight of the experimental animals was found. The animal experiments were approved by the Ethics Committee of Renmin Hospital of Wuhan University (approval number: WDRM-20200301C) and performed according to the AAALAC and the ARRIVE guidelines (https://arriveguidelines.org/, accessed on 10 February 2023) (https://www.aaalac.org/, accessed on 10 February 2023). All methods are reported in accordance with ARRIVE guidelines. During the surgery and the euthanasia, we have employed anesthesia consistent with the commonly accepted norms of veterinary best practice.

### 2.6. Construction of Circ_22232 Knockdown Mice

Two AAV (adeno-associated virus) vectors, AAV serotype 9 (AAV-CMV bGlobin-EGFP-empty) and AAV-CMV bGlobin-EGFP-circ_22232 Si (AAV-circ_22232 Si), were constructed by GeneChem Co., Ltd. (Shanghai, China). AAV-CMV bGlobin-EGFP-empty functioned as the blank control (AAV-circRNA Con). In addition, AAV-circ_22232 Si AAV or AAV-circRNA Con was microinjected into the left lateral ventricle of C57BL/6J mice at a rate of 0.2 µL/min. The MCAO was conducted 3 weeks after receiving AAV.

### 2.7. Middle Cerebral Artery Occlusion (MCAO) Model

Three weeks after receiving AAV, the MCAO surgery was conducted as previously described [16]. Briefly, mice were given 2% isoflurane in oxygen for 3 min, followed by 1.0–1.5% isoflurane in 70% N_2_O and 30% O_2_, and anesthesia was maintained by a small-animal anesthesia system. A midline neck incision was made to separate the left external carotid artery (ECA) and common carotid artery (CCA). After temporarily blocking the common and internal carotid arteries (ICA), a silicone rubber monofilament was inserted through the arteriotomy into the ECA and slowly reached the beginning of the middle cerebral artery (MCA) through the left ICA. After 1 h of occlusion, we removed the monofilament and permanently ligated the ECA. For the sham group, only the monofilament was inserted into the mice to block the MCA, and then the monofilament was removed, and blood flow was rapidly restored.

### 2.8. Schematic Mode

As described in Supplementary Figure S1, to study mRNA gene expression, we used 10 mice divided into two groups: a 2-day post-MCAO group and a sham control group (n = 5 in each group). To study the function and histology, we used 64 mice, divided into four groups randomly: sham control group, MCAO only group, MCAO + circ_22232 Con group, MCAO + circ_22232 Si group (*n* = 16 in each group). In each group, we tested the enzyme-linked immunosorbent assay (*n* = 5), brain water content (*n* = 5), 2,3,5-triphenyl tetrazolium chloride staining (*n* = 5), immunohistochemical (IHC), and hematoxylin-eosin (HE) staining (*n* = 3). To evaluate the neurological function of the circ_22232, we used 30 mice, randomly divided into 3 groups: MCAO only group, MCAO + circ_22232 Con group, and MCAO + circ_22232 Si group (*n* = 10 in each group).

### 2.9. Modified Neurological Severity Score (mNSS)

We applied the mNSS test to estimate the condition of these mice at different times after MCAO [17]. A higher score (scale from the highest score of 14 to the lowest score of 0) indicated more severe neurological impairment. Two observers scored the animal and provided their independent assessments in each group.

### 2.10. Triphenyl Tetrazolium Chloride (TTC) Staining

Mice were humanely euthanized within 24 h after MCAO. The euthanasia was conducted with a rapid intravenous injection of sodium pentobarbital by 75 mg per kg of body weight [18]. The brain was then incubated in a 2% TTC solution for 30 min at 37 °C before being transferred to a 4% paraformaldehyde solution and fixed for one day. After capturing digital images, the degree of infarction in each section was measured with Image J software.

### 2.11. Brain Water Content Analysis

Within 24 h after MCAO, we retrieved the brains. The wet weights of the left and right hemispheres were then determined. To acquire the dry weight, the brains were cooked at 75 °C for 48 h. The brain’s water content was then determined as [(wet weight − dry weight)/wet weight] 100%.

### 2.12. Quantitative Real-Time (PCR)

To extract RNA from tissues, the TRIzol reagent was utilized (Thermo Fisher Scientific, Massachusette, USA). For qRT-PCR, the SYBR Green system and specific primers were utilized (Supplementary Table S1). The qPCR experiments were performed at 95 °C for 10 min, followed by 45 cycles at 95 °C for 5 s, 60 °C for 30 s, and 72 °C for 25 s. For the outcomes of the control and experimental groups, the relative Ct method was utilized, with GAPDH (glyceraldehyde-3-phosphate dehydrogenase) serving as an internal reference.

### 2.13. Enzyme-Linked Immunosorbent Assays (ELISA)

Mice were anesthetized and transcardially perfused with phosphate-buffered saline (PBS, pH 7.4, 4 °C) 24 h after MCAO. Brain infarct zones were promptly collected, homogenized with 200 mg/mL of 0.9% balanced salt solution (BSS), and centrifuged at 12,000 rpm. The supernatant was then collected and kept at −80 °C. The quantities of interleukin 6 (IL-6), tumor necrosis factor alpha (TNF-α), interleukin 4 (IL-4), interleukin 1 (IL-1), and interleukin 10 (IL-10) in brain tissue lysates were determined with commercial ELISA kits (Schmallenberg Virus Antibody Test Kit, IDEXX, Westbrook, ME, USA). The ultimate concentration of cytokines is determined by the absorbance standard curve as described by the manufactory protocol.

### 2.14. Hematoxylin-Eosin Staining (H&E Staining)

Three mice per group were anesthetized and perfused with PBS (pH 7.4, 4 °C), followed by 4% paraformaldehyde solution (4 °C). After we removed the brains and post-fixed them in a 4% formaldehyde solution at 4 °C for 24 h, they were dehydrated and embedded in paraffin. These samples were then cut into 4 μm thick slices. These sections were de-paraffined and then rehydrated in decreasing ethanol concentrations and stained with H&E solution according to the manufacturer’s protocol (Sigma-Aldrich, St. Louis, MO, USA).

### 2.15. Immunohistochemistry

Coronal sections (4 μm thick) were prepared, deparaffinized, and rehydrated as described above. We fixed the tissues with 4% paraformaldehyde, embedded them in paraffin, and sectioned them. After rehydration, sections were treated with 3% H_2_O_2_ for 10 min and blocked with 1% bovine serum albumin for 1 h. Tissues were then incubated with anti-CD11b rabbit pAb (primary antibody) (1:400; #GB11105; proteintech), followed by the secondary antibody with horseradish peroxidase. The tissue was then developed in 3,3’-diaminobenzidine (DAB) and counter-stained with hematoxylin.

### 2.16. Statistical Analysis

The R (version 4.0.2) was applied to statistical analyses. Heat maps were plotted with the R package “pheatmap”. The volcano plot was drawn with the R package “Enhanced Volcano”. The survival curves were plotted with R package “survminer”. The GO chord plot was conducted with the R package “Goplot”. The Pearson correlation matrix was generated with the R package “corrplot”. Data were expressed as mean ± standard deviation. The *t*-test was conducted to assess differences between groups. A *p*-value < 0.05 was considered statistically significant.

## 3. Results

### 3.1. Sequencing Data Summary and DEG Analysis

**Figure 1:**
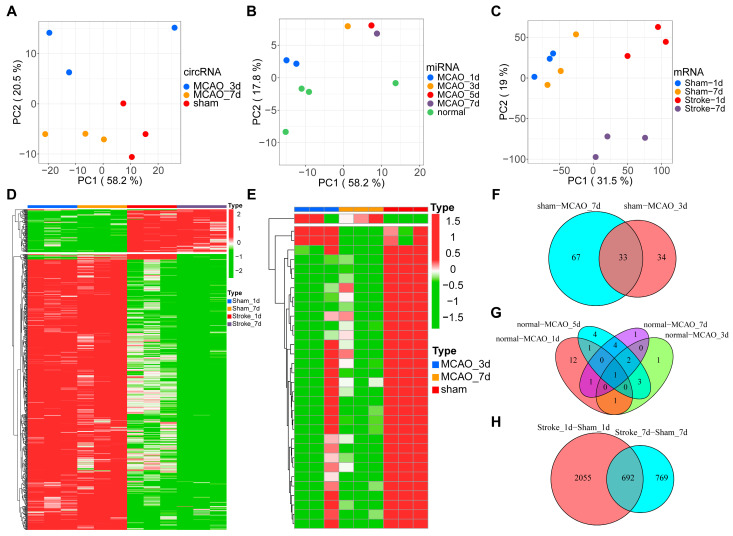
Differentially expressed genes involved in the pathophysiology of MCAO. (**A**–**C**) Principal component analysis investigates the distributions of circRNA (**A**), miRNA (**B**), and mRNAs (**C**). (**D**,**E**) Heatmap of DEGs about mRNAs (**D**) and circRNAs I between several different clusters; the rows indicate the expression levels of genes, while the columns indicate each sample. The shade of red means the levels of upregulated genes, while the shade of green means the levels of downregulated genes. (**F**–**H**) The common parts of the DEGs in circRNA (**F**), miRNA (**G**), and mRNA (**H**) among several groups were demonstrated by the Venn diagrams.

Principal component analysis indicated that the 9 circRNA samples were separated into three distinct groups (Figure 1A), the 9 miRNA samples were divided into five distinct groups (Figure 1B), and the 12 mRNA samples were divided into four distinct groups (Figure 1C). Data from the heatmap showed 692 differential expressed mRNAs and 33 differential expressed circRNAs (Figure 1D,E). Statistical analysis of circRNA showed that 100 differential circRNAs were identified in the brain on day 3 post-MCAO. This trend continued with 67 differential expressed circRNAs identified on day 7 post-MCAO. When two datasets were compared, 33 circRNAs were found to be identical in each (Figure 1F). Statistical analysis of miRNAs revealed that on the first day after MCAO, 16 different miRNAs were found in the brain; 8 different miRNAs on the third day after MCAO; 15 different miRNAs on the fifth day after MCAO; and 9 different miRNAs on the seventh day after MCAO. Four miRNAs were identical at four different time points (Figure 1G). On the first day, data from mRNA sequencing revealed that 2747 genes in the MCAO model were differentially regulated. On day 7, 1461 genes were found to be differently regulated. The integration of the two results showed 692 commonly identified genes (Figure 1H). The volcanic figure depicted individual DEGs from the mRNA and circRNA categories (Figure S2A,B). Importantly, far more genes were upregulated than downregulated following MCAO.

### 3.2. GO Enrichment and KEGG Pathway Analysis

**Figure 2:**
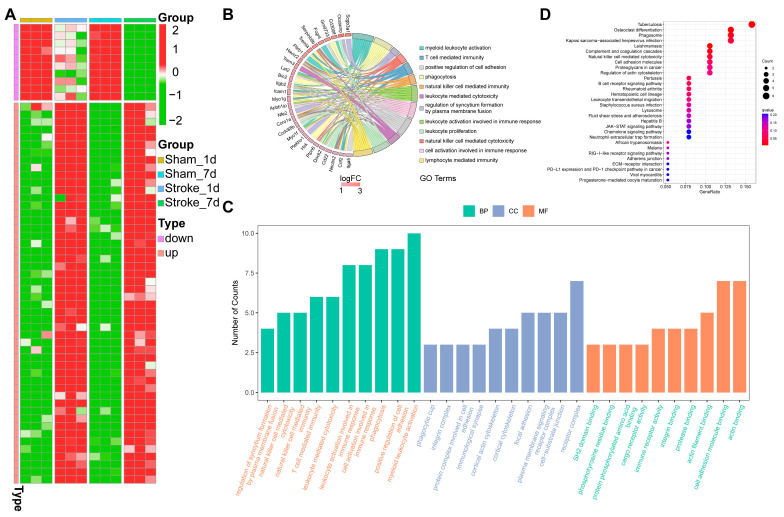
Gene enrichment analysis shows the relationship between our DEGs and immunity. (**A**) The heatmap shows the top 50 upregulated and top 10 downregulated DEGs. (**B**) Choral graph exhibits the correlations between 28 genes and 12 GO terms. The bottom bar indicates the log-foldchange values of these genes. (**C**) The top 10 function enrichment terms of biological process, cellular component, and molecular function. (**D**) KEGG analysis through analyzing these DEGs.

Figure 2A depicts the heatmap of the top 50 upregulated mRNAs and the top 10 downregulated mRNAs. The upregulated mRNAs were enriched in immunologic reactions (natural killer cell-mediated cytotoxicity, natural killer cell-mediated immunity, etc.) and activating immune response (cell activation involved in immune response, leukocyte activation involved in immune response, etc.); cellular component and molecular function showed to be association with inflammation (Figure 2B,C). The chemokine signaling pathway, JAK-STAT pathway, and neutrophil extracellular trap formation were all activated after MCAO in the KEGG pathway analysis (Figure 2D). All of these indicate that the DEGs were linked to the neuroinflammation in mice MCAO modes.

### 3.3. Coexpression Analysis of CircRNA Related to Inflammation

**Figure 3:**
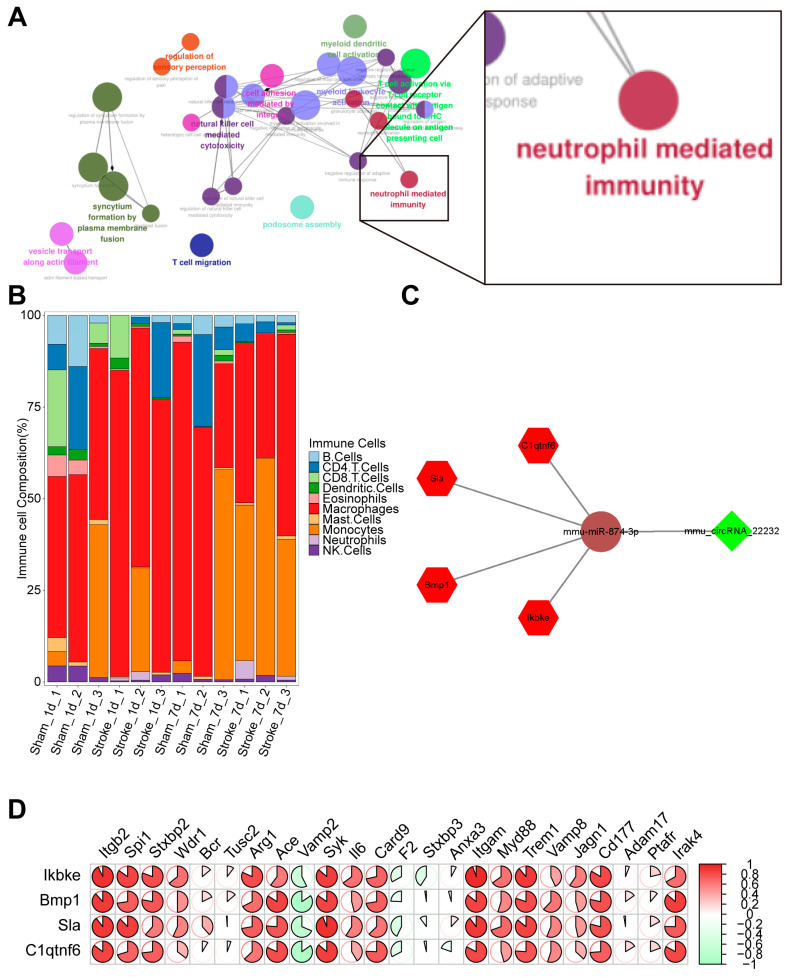
Screening out circRNA related to neuroinflammation promotions. (**A**) The network demonstrates the correlations between several Go terms by the ClueGO function of Cytoscape software; the right part indicated these DEGs were significantly related to neutrophil-mediated immunity. (**B**) The immune cells infiltration analysis by seq-ImmuCC. (**C**) Coexpression network of mRNA (red), miRNAs (brown), and circRNAs (green), lines between genes represent correlation. (**D**) The heatmap demonstrates the relationships between 4 hub genes (row names) and the neutrophil-mediated immunity-related genes (column names). The area and shade of the pies indicate the size of correlation values. The red indicates a positive relationship, while the green indicates a negative relationship.

To identify the circRNAs that regulate inflammation, regulatory network analysis of enriched GO terms was first performed by ClueGO. The result showed that inflammation was associated with neutrophil activation (Figure 3A). Next, we built a PPI network with key mRNAs involved in the activation of neutrophils (Figure S3). To further explore the dynamic changes of neutrophils in mice MCAO modes, we estimated the relative proportion of different immune cell subtypes in cerebral tissue by seq-immuCC [15]. The MCAO group exhibited a progressive enhancement in the proportion of neutrophils over the time course of 1 and 7 days post-MCAO compared to the control group (Figure 3B). We then used the Starbase database to obtain prediction files for circRNA-miRNA and miRNA-mRNA interaction and performed the integration analysis with previously obtained differentially expressed mRNA, circRNA, and miRNA to plot the interaction network (Figure 3C). Finally, we also plotted the correlation heat map of the four central genes that were strongly correlated with neutrophil-mediated immune response (Figure 3D).

### 3.4. Verification of Potential circRNA_22232/miR-874-3p/Bmp1 Axis in Neuroinflammation

**Figure 4:**
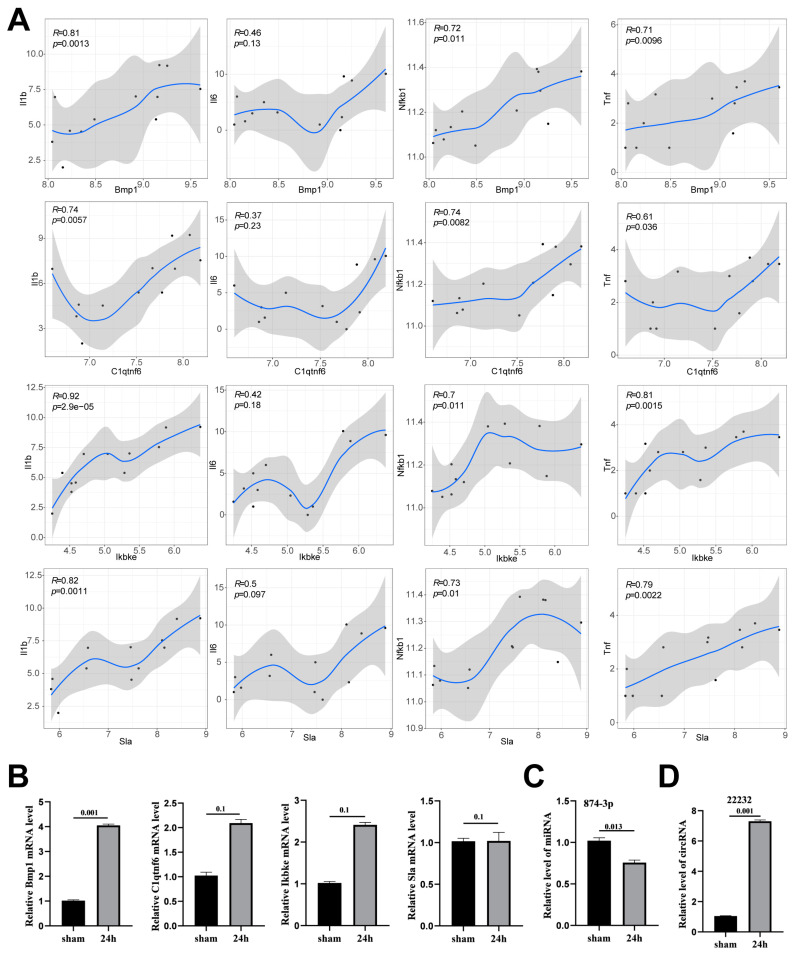
Verification of circRNA_22232/miR-874-3p/Bmp1 axis related to neutrophil activation after ischemic stroke. (**A**) The correlation analysis among 4 hub genes (Bmp1, C1qtnf6, Ikbke, and Sla) and 4 proinflammatory genes (Il1b, Il6, Nfkb1, and Tnf), based on the mRNA expression levels of them. (**B**) The mRNA expressions from the cortex of different groups of mice were measured by qPCR. Data are presented as the mean ± SEM, *n* = 5, Mann–Whitney test. (**C**,**D**) The expression of circ_22232 and miR-874-3p in MCAO was measured with qPCR. The data represent the mean ± SEM. *n* = 5 in each group.

During the initial phase of IS, activated immune-related cells may produce a series of proinflammatory cytokines that promote neuroinflammation and further exacerbate brain injury. Therefore, we subsequently analyzed the association between the mRNAs of proinflammatory cytokines secreted by immune cells and the expression levels of three potential mRNAs. The results showed that Bmp1, C1qtnf6, Ikbke, and Sla were positively associated with the expression of proinflammatory cytokines (Il1b, Il6, Nfkb1, and Tnf) (Figure 4A). Furthermore, to confirm these coexpression results, we established the mouse model that was subjected to 1 h MCAO and 24 h reperfusion, and the RNA expression levels were detected by qPCR. The result indicated that Ikbke, Sla, and C1qtnf6 did not differ significantly, but the Bmp1 differed significantly between the control and MCAO groups (Figure 4B). Consistently, the miR-874-3p level was significantly reduced (Figure 4C), while the circRNA_22232 level was upregulated in the MCAO model (Figure 4D). Combined with the results of qPCR as well as bioinformatics analysis, these data indicate that the circRNA_22232/miR-874-3p/Bmp1 axons may be involved in neuroinflammation after IS. Therefore, we decided to further investigate the specific role of circRNA_22232.

### 3.5. Circ_22232 Knockdown Reduced Ischemic Brain Injury and Release Proinflammatory Cytokine

**Figure 5:**
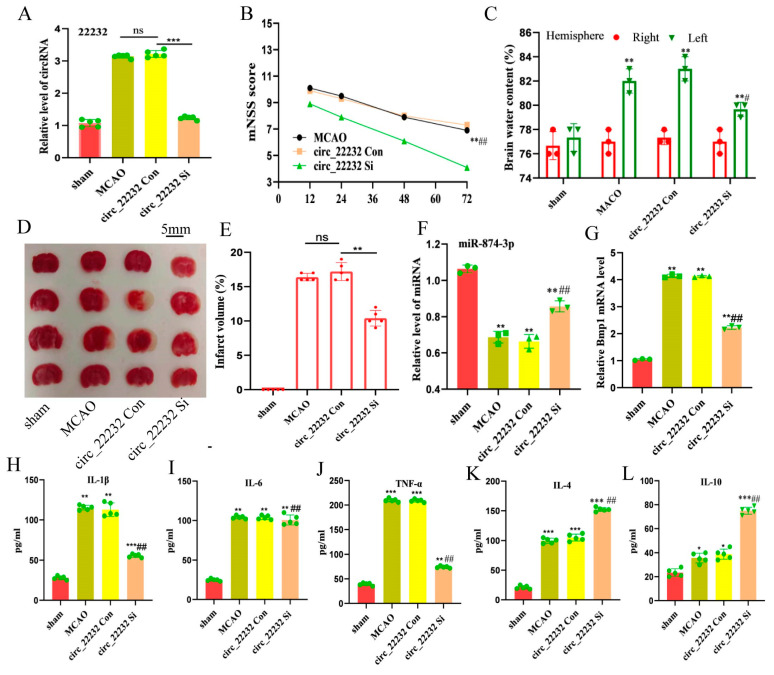
Circ_22232 knockdown dropped the infarct volume and proinflammatory cytokines after MCAO. (**A**) The mRNA of circ_22232 was measured with qPCR. Data are presented as the mean ± SEM, *n* = 5, *** *p* < 0.001 versus circRNA Con, ns *p* > 0.05 versus MCAO, Mann–Whitney test. (**B**) Time course of mSSs. Data are presented as the mean ± SD, *n* = 5, ** *p* < 0.01 versus circRNA Con, ## *p* < 0.01 versus MCAO. Mann–Whitney test. (**C**) The severity of brain edema was measured. Bars indicate the mean ± SD, *n* = 3, ** *p* < 0.01 versus sham group, # *p* < 0.05 versus circRNA Con group, *t*-test. (**D**) TTC-stained brain slices in sham, MCAO, circRNA Con, and circ_22232 Si mice at 24 h after MCAO. (**E**) Comparison of the percentages of infarct volume between each group of mice. *n* = 5, ** *p* < 0.01 versus circRNA Con, Mann–Whitney test. (**F**,**G**) The mRNA of miR-874-3p and Bmp1 were measured with qPCR. Data are presented as the mean ± SD, *n* = 5, ** *p* < 0.01 versus circRNA Con, ## *p* < 0.01 versus MCAO. Mann–Whitney test. The protein levels of IL-1β (**H**), IL-6 (**I**), TNF-α (**J**), IL-4 (**K**), and IL-10 (**L**) in the infarcted cortex at 24 h after MCAO were detected with ELISA. Data represent the mean ± SD. *n* = 5. *** *p* < 0.001, ** *p* < 0.01, and * *p* < 0.05 versus sham group; ## *p* < 0.01 versus circRNA Con, *t*-test.

To further clarify the role of circ_22232 in mice MCAO model, Mice were divided into four groups: sham control group, MCAO only group, the MCAO plus circ_22232 Con, and the MCAO plus circ_22232 Si. The circ RNAs AAV were microinjected into the lateral ventricles of the mice. The knockdown of circ_22232 was first confirmed (Figure 5A). After confirming that the circ_22232 could be stably knockdown in vivo, the mice MCAO was then performed. We then assessed the neurological function scores of different groups. It revealed that the mice’s neurological function was considerably compromised in the circ_22232 Con group, and the circ_22232 Si could alleviate the brain injury after MCAO (Figure 5B). Interestingly, the circ_22232 Si group mice exhibited less cerebral edema (Figure 5C), as well as the smaller infarct volume revealed with TTC staining (Figure 5D,E). In addition, the miR-874-3p level was upregulated, while Bmp1 was reduced when knocking down circ_22232 in the MCAO mouse model (Figure 5F,G). This finding was consistent with previous results. Next, we looked at whether circ_22232 knockdown could regulate the levels of the inflammatory cytokines. Three proinflammatory cytokines—IL-1, IL-6, and TNF-α (Figure 5H–J), as well as two anti-inflammatory cytokines—IL-4 and IL-10 (Figure 5K,L)—are detected with ELISA. With the circ_22232 knockdown, the levels of IL-1β and TNF-α decreased, while the levels of IL-10 and IL-4 increased. All of these promising results suggest that circ_22232 knockdown could be able to reduce the inflammatory response, hence altering brain injury.

### 3.6. Circ_22232 Knockdown Reduced Ischemic Brain Injury and Activated Neutrophils

**Figure 6:**
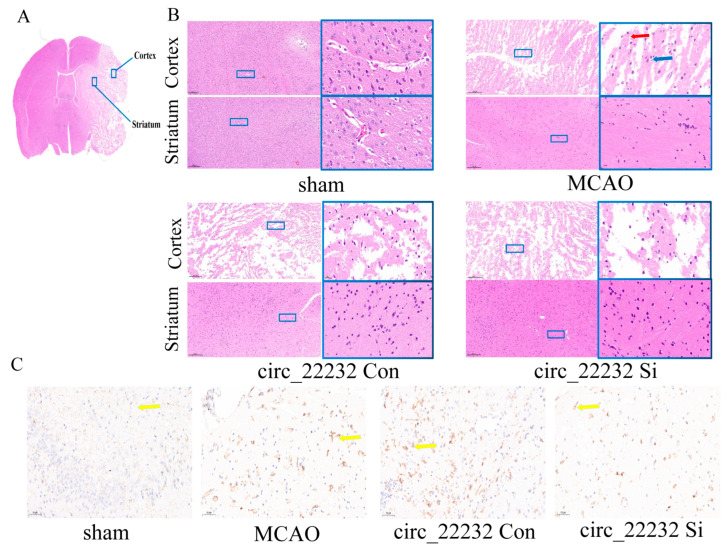
Circ_22232 knockdown attenuated cerebral ischemic injury and reduced neutrophil activation in mice MCAO mode. (**A**) Representative images of histological staining of striatum and cortex in mice MCAO modes. Coronal sections were stained with H&E (**B**) and immunohistochemistry of anti-CD11b (**C**). Scale bar, 20 μm. The blue box shows the enlarged area.

We then observed the pathological changes in the striatum and cortex by histology (Figure 6A). Morphological alterations were explored with HE staining. The cortex and striatum of circ_22232 knockdown mice contained significantly fewer vacuolated cytoplasm and pyknotic nuclei (Figure 6B). After MCAO, HE and immunohistochemical staining of CD11b revealed the presence of neutrophils and morphology. We observed that neutrophils had shorter ramifies and larger cell bodies in the circ_22232 knockout group compared with the control. Importantly, the circ_22232 knockdown group showed significantly fewer active neutrophils (Figure 6C).

## 4. Discussion

Cerebral ischemia triggers a series of pathological and metabolic reactions that lead to brain tissue injuries, such as cerebral infarction and neuronal death [19]. Neuroinflammation is an inflammatory response that functions as an adaptive response to tissue damage [20]. The transiently upregulated inflammatory process shows a protective effect in some respects, but as the inflammatory stimulus continues to expand, a series of inflammatory cascades occur, leading to secondary brain damage [5]. Thus, the inflammatory response plays a crucial role in IS, and its regulatory mechanism deserves further investigation.

CircRNAs are expanded substantially throughout neuronal differentiation and maturation and are prevalent in the brain. According to mounting evidence, CircRNAs are more sensitive to cerebral ischemia; they regulate biological metabolism, intercellular communication, and the binding of proteins, ions, and nucleic acids, and their functional changes can affect physiological and pathological processes after stroke [21]. It should be emphasized that circRNAs reflect many genes in the gene regulatory network and can function as biomarkers due to their extremely stable characteristics [22]. However, only a few circRNAs have been studied, and it is mostly unknown how they contribute to cerebral ischemia. In our study, we applied bioinformatics analytical methods to analyze these high-throughput sequencing data. We identified for the first time circRNA_22232 associated with inflammation. In addition, we demonstrated that circRNA 22232/miR-874-3p/Bmp1 pathway in IS may be immune-related and linked to neutrophil activation in vivo and that circRNA_22232 knockdown can have an anti-inflammatory effect.

Inflammation plays an indispensable role in maintaining the steady state of tissues under adverse conditions such as ischemia and hypoxia. Inflammation starts minutes after the onset of ischemic stroke, becomes the major pathogenic mechanism within hours, and may persist for a long time [23]. Currently, several reports suggest that circRNAs are involved in the pathogenesis of IS by modulating inflammatory response [24]. On the one hand, circRNAs may have an anti-inflammatory role in IS. The nature of BBB is a vital structure to maintain the homeostasis of the central nervous system. Disruption of the BBB may result in neuronal injury, amplification of the neuroinflammatory response, and peripheral inflammatory cell infiltration. Moreover, extracellular vesicles that contain proteins, lipids, and nucleic acids can cross the BBB [25]. Constructing extracellular vesicles containing circSCMH1 and injecting them into mice through the tail vein reduced glial cell activation and peripheral immune cell infiltration, thus promoting functional recovery after IS [26]. On the other hand, circRNAs may have a proinflammatory role in IS. Astrocytes are regulators of neuroinflammation, and activation of astrocytes can worsen the inflammatory responses of the brain and neuronal injury. Thus, astrocyte inhibition could protect the brain from damage. CircHECTD1 is an endogenous miR-142 reservoir that inhibits miR-142 activity. For the cerebral tissue of mice MCAO mode, miR-142 was markedly downregulated while circHECTD1 was substantially increased. Increased circHECTD1 expression has been linked to higher levels of C-reactive protein, proinflammatory cytokines, and National Institutes of Health Stroke Scale scores in the peripheral blood of AIS patients, suggesting that circHECTD1 may serve as a biomarker of inflammation in IS [27]. In our research, animal studies were used to better understand the anti-inflammatory role of circRNA_22232 in early IS. Furthermore, we first showed the dynamic transcriptome changes in neutrophils and circRNA_22232 at multiple time points after MCAO by looking at sequencing data from the cerebral cortex.

In most cases, inflammation is associated with tissue repair and has positive consequences, including phagocytosis of dead cells and debris. However, overreacting and overheating inflammation in the case of IS may result in neurotoxic effects that aggravate the underlying disease state. The activation of immune-related cells is the first step in this process [28]. Macrophages, neutrophils, and lymphocytes can infiltrate the brain after ischemia without obstructing BBB [29]. Since neutrophils are the primary source of the free oxygen radicals linked to direct neuronal death, they make up a significant portion of all cell populations covering the injured tissue during the first 1 and 3 days after ischemia [30], which has been widely accepted as key contributors in inflammatory brain injury. In addition to neutrophils, endothelial cell dysfunction and the release of matrix metalloproteinase 9 lead to BBB disruption [31]. Several reports have indicated that circRNAs play a major role in IS-induced inflammation [32]. In our study, the proportion of neutrophils was significantly higher after MCAO. Neutrophils are one of the forerunners of ischemic lesions in multiple organs. Neutrophils perform intricate activities inside stroke lesions after being called to action by neutrophil-attracting chemokines. The phenotypes of neutrophils are diverse, just as those of microglia. It showed that the N1 phenotype enhanced cerebral inflammation while N2 neutrophil contributed phagocytic activities that encouraged inflammatory resolution within the stroke [33]. Furthermore, the development of neutrophil extracellular traps (NETs), which are neutrophils’ extracellular chromatin-containing products, may exacerbate neuronal injury [34]. Therefore, it is important to think about modulating neutrophil activity as a potential treatment for IS. Studies have also shown that reducing neutrophil accumulation at any stage of IS prevents the development of neuroinflammation [35], which supports the conclusion that the potential role of circ_22232 is associated with a reduction in neutrophil accumulation in stroke lesions. In addition, the anti-inflammatory factors IL4 and IL10 were elevated when circ_22232 was knocked down. However, we did not test whether the specific subtype of neutrophils belongs to N1 or N2. Based on the levels of proinflammatory cytokines detected by ELISA, it can be predicted that circ_22232 directs the neutrophil polarization toward the N2 phenotype. Therefore, it suggests that preventing neutrophil invasion and accelerating neutrophil clearance can decrease the number of neutrophils in stroke lesions to better predict the outcome. It also shows that neutrophils with the N2 phenotype support a beneficial inflammatory response.

It has been reported that with the activation of microglia via Janus kinase JAK1 signaling, interferon γ activates the STAT1 factor and increases the level of proinflammatory cytokines such as TNF-α, IL-23, IL1β, IL-12, chemotactic factors, and reactive oxygen species [36]. It also shows that microglia polarization to M1 is associated with the activation of NF-κB [37]. Similar to macrophages, neutrophils respond to all trans-retinoic acid treatments and develop toward the N2 phenotype in the context of STAT1 signaling [38]. Following IS, as an important transcription factor, NF-κB can co-activate downstream inflammatory factors such as TNF-α through complex regulation [39] and participate in the inflammatory cascade and oxidation following MCAO/R [40]. Furthermore, inflammatory-associated cell signaling cascades, such as the mitogen-activated protein kinase (MAPK) pathway, have been implicated in the pathogenesis of IS [41]. In our study, the potential circRNA function and biological pathways were predicted by KEGG analysis. The result showed that activation of NF-κB and JAK-STAT pathway were consistent with these findings. Thus, these aberrantly expressed circRNAs associated with inflammation and immunity are involved in many pathophysiological processes in IS. These circRNAs may serve as biomarkers and therapeutic targets.

Unfortunately, several limitations hinder the clinical application of circRNA-related immunoregulatory therapies. First, there is a systematic bias regarding the deconvolution analysis of immune cell proportion. Single-cell RNA sequencing (scRNA-seq), which possesses a higher resolution, should be performed to fully elucidate the circRNA [42]. Furthermore, the upstream of circRNA is regulated by histone modification, methylation, and variation in gene copy number. There are many difficulties in predicting which molecules upstream of circRNA_22232 are regulated. Second, there is a divergence between experimental and clinical stroke. The efficacy of immunoregulatory therapy in animals is based on the MCAO model, which cannot fully replicate the clinical situation. Most patients with ischemic stroke experience permanent ischemia without reperfusion. Thus, it is necessary to explore the transcriptomic data of human brain tissue that suffered from IS. Finally, we were only exploring the immunoinflammatory role of circRNA_22232 in IS. The potential role of circRNA_22232/miR-874-3p/Bmp1 pathway should be further investigated in future studies.

## 5. Conclusions

In conclusion, our findings revealed potential immune-associated circRNA_22232, which provides a novel direction to elucidate the molecular mechanism of IS and explore potential therapeutic targets. It was found that neutrophil-mediated immune-associated circRNA_22232 was upregulated, and the knockdown of circRNA_22232 prevented ischemic brain injury associated with neuroinflammation. Therefore, circRNA_22232 may be a candidate for the treatment of cerebral ischemia caused by neuroinflammation.

## Data Availability

The datasets generated and analyzed during the current study are available in the GEO database (https://www.ncbi.nlm.nih.gov/geo/, accessed on 8 January 2023). And, the accession numbers are GSE46266, GSE131193, and GSE196448. The raw data supporting the conclusions of this article will be made available by the first author (Zheng Sun) without undue reservation.

## Supplementary Materials

The following supporting information can be downloaded at: https://www.mdpi.com/article/10.3390/brainsci13091283/s1. Figure S1: Flowchart of our study. Figure S2: (A–D) Volcano plots show the differential expression of circRNAs MACO_3d (A) and MACO_7d (B) as well as mRNAs MACO_1d (C) and MACO_7d (D). Figure S3: PPI network exhibits the relationships of DEGs involved in neutrophil-mediated immunity. Supplementary Table S1: Primer sequence of qPCR.
