# Supplementary material for: Transcriptome-Wide Analysis of Neutrophil-Related Circ_22232 in Neuroinflammation from Ischemic Stroke Mice
Source: Brain Sci. 2023 Sep 4;13(9):1283. doi: 10.3390/brainsci13091283 (PMC10526308; doi:10.3390/brainsci13091283)
Supplement: Supplementary file 1 [file brainsci-13-01283-s001.zip › Supplemental Table S1.pdf]

Table S1 Primer Sequence of qPCR

Gene	Sequence (5′ to 3′)
Circ_22232	F: GAGCCGAATAAGGGAAGAAAGG<br>R: CACTGAGATGGTCTTGGCACTT
miR-874-3p	F: GAACTCCACTGTAGCAGAGATGGT<br>R: CATTTTTTCCACTCCTCTTCTCTC
Bmp1	F: TGGCCGACTACACCTATGA<br>R: GGAGGACTTACGAGCTGTGT
C1qtnf6	F: GAAAGGGTCTTTGTGAACCTTGA<br>R: CTGCGCGTACAGGATGACAG
Ikbke	F: GAGAAGTTC GTCTCGGTCTATGG<br>R: TGCATGGTACAAGGTCACTCC
Sla	F: ATGGGAAGTTTGTCCAGCAGAGGG<br>R: AGCATCATCCAAGGGGTCCTCAGC

（Denaturation at 92~96℃,Annealing at 67℃,CAmplification at 72℃.）
