# Supplementary figures and images for: Transcriptome-Wide Analysis of Neutrophil-Related Circ_22232 in Neuroinflammation from Ischemic Stroke Mice
Source: Brain Sci. 2023 Sep 4;13(9):1283. doi: 10.3390/brainsci13091283 (PMC10526308; doi:10.3390/brainsci13091283)

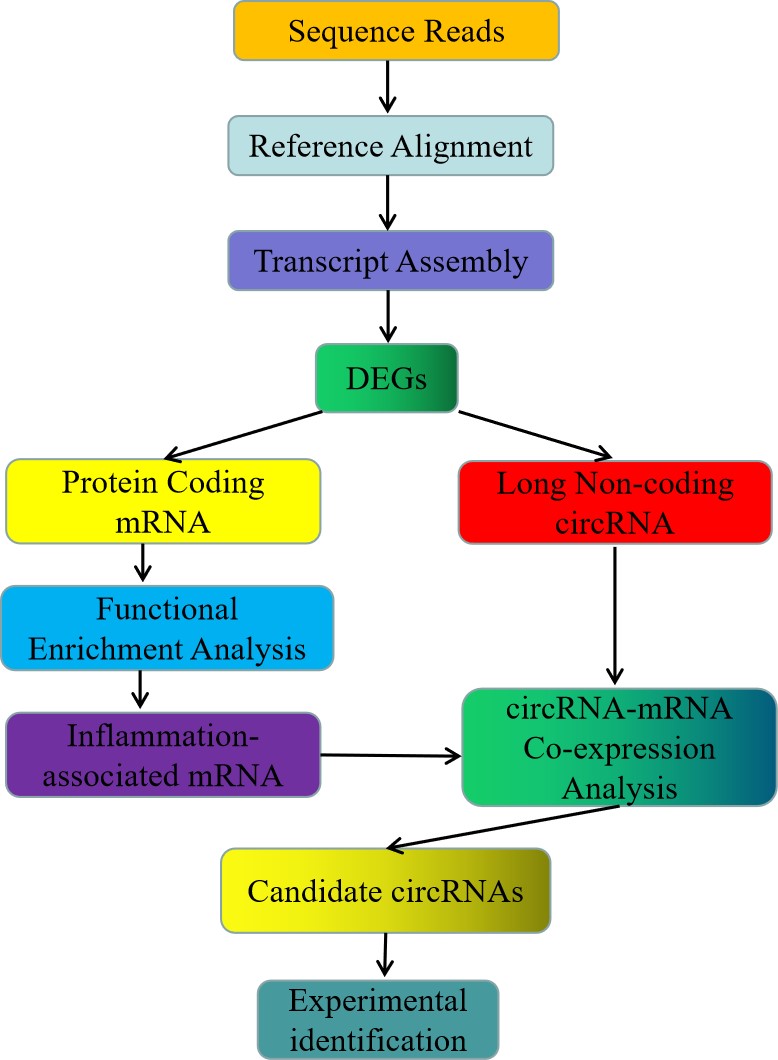

Supplement: Supplementary file 1 [file brainsci-13-01283-s001.zip › Figure S1.jpg]

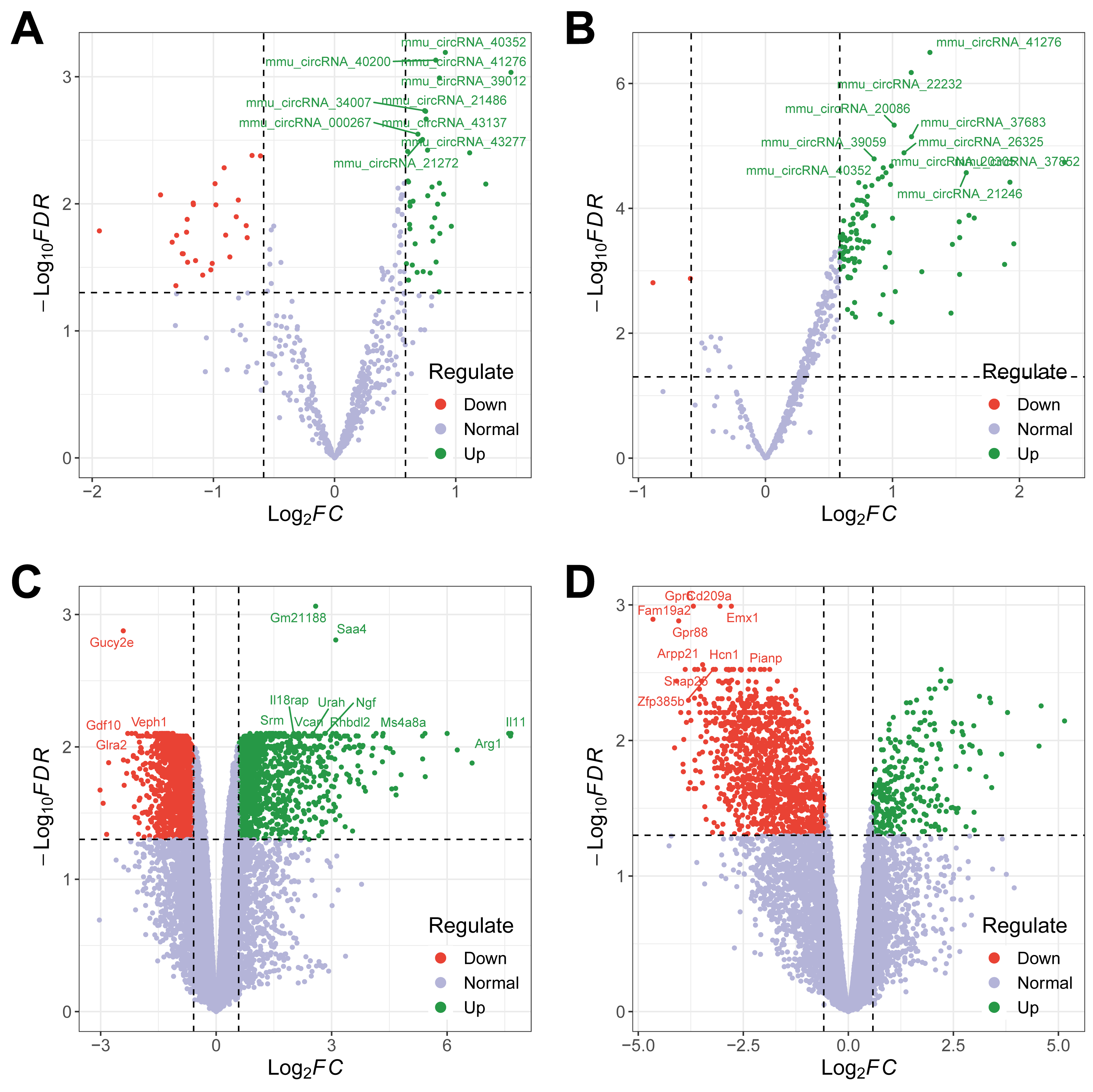

Supplement: Supplementary file 1 [file brainsci-13-01283-s001.zip › Figure S2.tif]

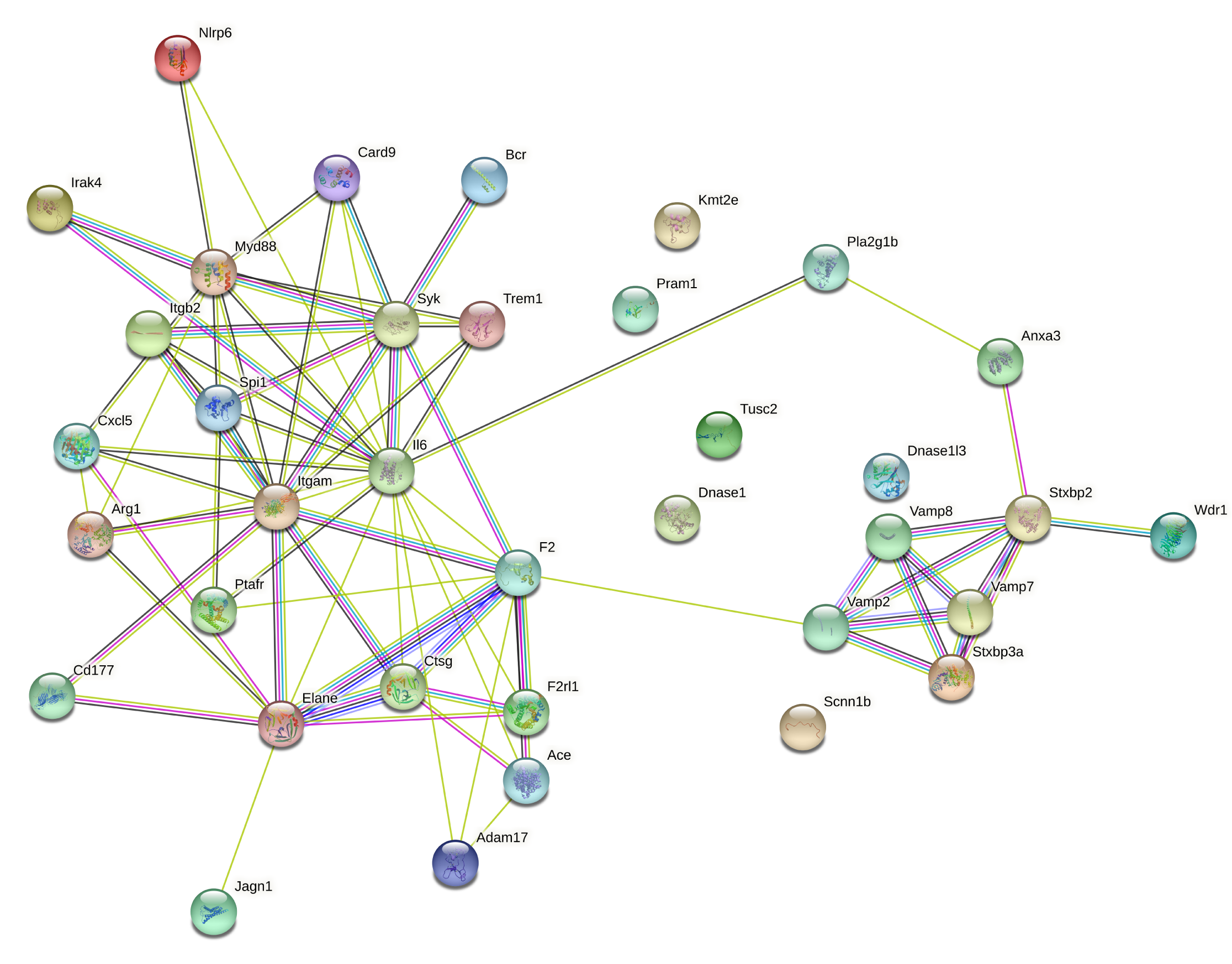

Supplement: Supplementary file 1 [file brainsci-13-01283-s001.zip › Figure S3.tif]
